# A Bio-Inspired Nanotubular Na_2_MoO_4_/TiO_2_ Composite as a High-Performance Anodic Material for Lithium-Ion Batteries

**DOI:** 10.3390/ma14020357

**Published:** 2021-01-13

**Authors:** Bo Yu, Zehao Lin, Jianguo Huang

**Affiliations:** Department of Chemistry, Zhejiang University, Hangzhou 310027, China; 21837072@zju.edu.cn (B.Y.); 11637057@zju.edu.cn (Z.L.)

**Keywords:** biomimetic synthesis, cellulose, layer-by-layer self-assembly, titania, sodium molybdate, lithium-ion batteries

## Abstract

A train of bio-inspired nanotubular Na_2_MoO_4_/TiO_2_ composites were synthesized by using a natural cellulose substance (e.g., commercial ordinary filter paper) as the structural template. The TiO_2_ gel films were coated on the cellulose nanofiber surfaces via a sol-gel method firstly, followed with the deposition of the poly(diallyldimethylammonium chloride)/Na_2_MoO_4_ (PDDA/Na_2_MoO_4_) bi-layers several times, through the layer-by-layer self-assembly route, yielding the (PDDA/Na_2_MoO_4_)_n_/TiO_2_-gel/cellulose composite, which was calcined in air to give various Na_2_MoO_4_/TiO_2_ nanocomposites containing different Na_2_MoO_4_ contents (15.4, 24.1, and 41.4%). The resultant nanocomposites all inherited the three-dimensionally porous network structure of the premier cellulose substance, which were formed by hierarchical TiO_2_ nanotubes anchored with the Na_2_MoO_4_ layers. When employed as anodic materials for lithium-ion batteries, those Na_2_MoO_4_/TiO_2_ nanocomposites exhibited promoted electrochemical performances in comparison with the Na_2_MoO_4_ powder and pure TiO_2_ nanotubes, which was resulted from the high capacity of the Na_2_MoO_4_ component and the buffering effects of the TiO_2_ nanotubes. Among all the nanotubular Na_2_MoO_4_/TiO_2_ composites, the one with a Na_2_MoO_4_ content of 41.4% showed the best electrochemical properties, such as the cycling stability with a capacity of 180.22 mAh g^−1^ after 200 charge/discharge cycles (current density: 100 mA g^−1^) and the optimal rate capability.

## 1. Introduction

In recent years, energy resources have been gradually dwindling due to the excessive exploitation. Lithium-ion batteries (LIBs) are a kind of environmentally friendly and rechargeable energy-storage devices, which acts as a pivotal part in human life, owing to their high energy densities, high columbic efficiencies, low self-discharge features, and extensive chemical potentials for the diverse design of electrodes [1]. They have been broadly applied in diverse fields containing intelligent mobile phones, mobile laptops, digital cameras, new energy vehicles, power grid energy storage, and so on [2,3,4]. A lithium-ion battery is usually composed of four parts: the positive and negative electrodes, the nonaqueous electrolyte, as well as the separator membrane. Among them, the energy storage performance is mainly decided by the electrode materials because the charges are stored on or in the electrodes. Accordingly, the requirement for the development of high-efficiency and long-term stable electrode materials for LIBs is crucial [5].

At present, the graphite-based materials have been widely used as the commercial anodes for LIBs based on the insertion and desertion mechanism. Unfortunately, the theoretical capacity of the graphitic carbon (372 mA g^−1^) is rather low to satisfy the impending need of high energy densities [5]. Therefore, immense efforts have been paid on the investigation of the high-performance anodic materials, especially the transition metal oxides, such as NiO [6,7], MnO [8,9], Mn_3_O_4_ [10], Fe_3_O_4_ [11,12,13], Fe_2_O_3_ [14], SnO_2_ [15], and V_2_O_3_ [16], etc. The metal oxides are outstanding electrode material candidates for lithium storage on account of their excellent electronic conductivities, larger theoretical capacities, as well as favorable diffusion abilities of lithium ions [17]. Specifically, Mo-based compounds have been investigated in large quantities due to the large capacities owing to the conversion reaction from Mo^6+^ to Mo^0^ with the transfer of six electrons [18,19,20,21,22,23]. In addition, Mo^6+^ is easily reduced, which causes the appearance of conversion type reactions at lower voltage vs. Li^+^/Li [20,22]. Mo-based oxides, dichalcogenide, and oxysalts have been investigated on their electrochemical properties as the anodic materials for LIBs, such as MoO_3_ [24,25], MoO_2_ [26,27], MoS_2_ [28,29], MMoO_4_ (M = Na, Fe, Ni, La, Zn, Ca) [21,30,31,32,33,34,35,36], and MMo_2_O_7_ (M = Na, Li, Ag) [18,19,20]. However, the electronic conductivities and long-term cycling performances of these materials remain to be improved. In order to overcome these shortcomings, reducing the size of the materials to the nanoscale is considered as an effective method to alleviate volume changes, increase active sites, accelerate charge conduction, and promote ion diffusion [37,38]. Besides, combining Mo-based materials with other materials (e.g., carbon, metal oxides) also increases the ionic and electronic conductivities and buffers the huge volumetric strain of the Mo-based materials, leading to the significant enhancement of their electrochemical performances [39,40]. Among all sorts of the molybdate salts, sodium molybdate has many advantages of a high theoretical specific capacity (780.6 mAh g^−1^) [21], low cost, as well as environmental friendliness [41,42,43]. Some of the sodium molybdate based materials were produced and applied as the anodic materials for LIBs, such as the carbon-coated α-Na_2_MoO_4_ nanoplate and Na_2_Mo_2_O_7_ nanocrystalline samples prepared via the sol–gel method [20,21], the Y_2_(MoO_4_)_3_ nanowires synthesized by the electrospinning technique [23], and so on. However, there were few researches concerning the development of the Na_2_MoO_4_-based materials with specific nanostructures as the anodic materials for LIBs previously.

The preparation of materials for rechargeable batteries using the bio-inspired synthesis routes is now a research hotspot [44,45]. Bio-inspired synthesis based on the layer-by-layer (LbL) self-assembly route serves as an efficient and environmentally friendly technique to produce materials with designed structures and functionalities, which offers an approach for the synthesis of the above-mentioned nanostructured Na_2_MoO_4_-based materials. Among the natural substances, natural cellulose substances are one of the most promising candidates due to the excellent properties of unique hierarchically macro-to-nano network structure, low cost, biocompatibility, and environmental friendliness [46]. Furthermore, the plentiful hydroxyl groups on the cellulose nanofiber surfaces are beneficial to the deposition of building blocks layer by layer, leading to the fabrication of nanostructured functional materials [46]. In those researches we have already done, different metal oxides or silicon based nanocomposites were fabricated by employing natural cellulose substances as the structural templates or carbon sources, which exhibited excellent electrochemical properties when applied as anodic materials for LIBs on account of their distinctive three-dimensional nanostructures derived from the premier cellulose substance and the cooperative effects of the constituents in the composites [25,47,48].

In this paper, a nanotubular Na_2_MoO_4_/TiO_2_ composite was yielded by using a crude cellulose substance (e.g., commercial ordinary filter paper) as the structural template on the basis of the LbL self-assembly route. These Na_2_MoO_4_/TiO_2_ nanocomposites absolutely duplicated the interwoven network structure of the premier cellulose substance, which were composed of TiO_2_ nanotubes deposited with varied contents of Na_2_MoO_4_ particles. When the nanocomposites were applied as the anodic materials for LIBs, they showed ameliorated electrochemical properties in comparison with the pure Na_2_MoO_4_ powder and pure TiO_2_ nanotubes, which were resulted from their extraordinary three-dimensionally interwoven structures and the cooperative effects betwixt the TiO_2_ and Na_2_MoO_4_ components.

## 2. Materials and Methods

### 2.1. Materials

Titanium (IV) n-butoxide (Ti (O^n^Bu)_4_) and Sodium molybdate (Na_2_MoO_4_) were obtained from J&K Chemical Ltd. (Beijing, China). Poly(diallyldimethylammonium chloride) (abbreviated as PDDA, typical MW = 200,000–350,000, 20 wt% aqueous solution) was purchased from Sigma–Aldrich (St. Louis, MO, USA). The quantitative filter paper was acquired from Hangzhou Xinhua Paper Industry Co. Ltd. (Hangzhou, China). Ethanol and toluene were gained from Sinopharm Chemical Reagent Co. Ltd. (Shanghai, China). The total chemicals were guaranteed reagents and directly employed without extra depuration. The utilized water was purged by a Milli-Q Advantage A10 system (Millipore, Bedford, MA, USA) with a specific resistance above 18.2 MΩ cm^−1^.

### 2.2. Preparation of the Na_2_MoO_4_/TiO_2_ Nanocomposites

**Scheme 1:**
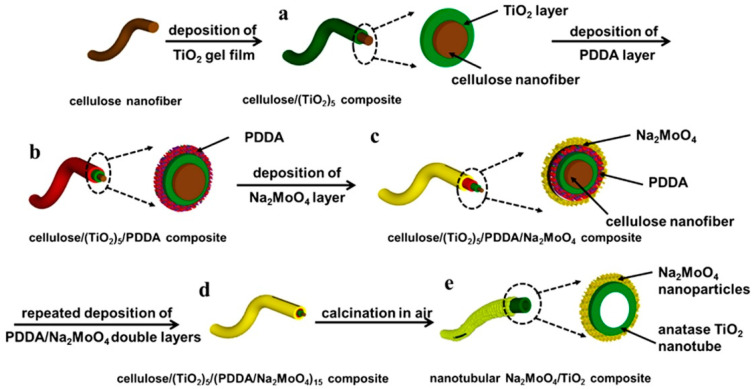
Schematic illustration of the synthetic processes for the nanotubular Na_2_MoO_4_/TiO_2_ composite derived from natural cellulose substance. (**a**) The titania–cellulose-nanofiber composite was prepared by the surface sol-gel process. (**b**) The PDDA/titania/cellulose-nanofiber composite was obtained by depositing the PDDA layer onto the titania–cellulose-nanofiber composite surface. (**c**) The Na_2_MoO_4_/PDDA/titania/cellulose-nanofiber composite was prepared by depositing the Na_2_MoO_4_ layer onto the PDDA/titania/cellulose-nanofiber composite surface. (**d**) The (PDDA/Na_2_MoO_4_)_15_/titania/cellulose composite was prepared by repeating the deposition of the PDDA/Na_2_MoO_4_ double layer 15 times. (**e**) The nanotubular Na_2_MoO_4_/TiO_2_ composite was obtained by the calcination of the as-deposited (PDDA/Na_2_MoO_4_)_15_/titania/cellulose composite sheet in air.

Scheme 1 presents the fabrication processes of the nanotubular Na_2_MoO_4_/TiO_2_ composites stemmed from the natural cellulose substance (e.g., commercial ordinary filter paper), which is composed of casually interwoven cellulose microfiber assemblies, that are formed by numerous cellulose nanofibers (Figure S1, Supplementary Materials). On the basis of our previous report [46], Ti(O^n^Bu)_4_ precursor was applied to deposit the ultrathin titania gel films onto the cellulose nanofiber surfaces of the filter paper via the surface sol-gel process. A slice of ordinary quantitative filter paper laid in the suction filtration was rinsed by ethanol firstly and dried with air stream for 15 min. The Ti(O^n^Bu)_4_ solution (10.0 mL, 100 mM in toluene/ethanol solvent (*v:v* = 1:1)) was added into the funnel for 3 min so that the precursor was adequately adsorbed on the cellulose nanofibers. Then, 10.0 mL of ethanol was added and filtered to remove the physically adsorbed Ti(O^n^Bu)_4_, followed with the filtration of 20.0 mL water to promote hydrolysis. After repeating the deposition cycle 5 times, the titania–cellulose-nanofiber composite with a negatively charged surface was successfully prepared (Scheme 1a). Subsequently, 20 mL of PDDA aqueous solution (1.0 g L^−1^ in water) was put in the filter funnel, and half of it was slowly suction-filtered off, while the rest was kept for 8 min. Then a small amount of PDDA solution was added and kept for another 8 min to make it thoroughly absorbed on the surfaces of the as-deposited titania gel layers by electrostatic interaction. Afterwards, 20.0 mL of water was filtered to wash away the unassembled reagent. Finally, the composite sheet was desiccated in airflow for 18 min, resulting in the PDDA/titania/cellulose-nanofiber composite (Scheme 1b). The Na_2_MoO_4_ layers were deposited close behind on the surfaces of the PDDA layers in the same way with various concentrations of the Na_2_MoO_4_ solution (0.02 M, 0.05 M, 0.1 M) to give the Na_2_MoO_4_/PDDA/titania/cellulose-nanofiber composites (Scheme 1c). The sedimentation of the PDDA/Na_2_MoO_4_ bi-layer was repeated 15 times, then the as-deposited (PDDA/Na_2_MoO_4_)_15_/titania/cellulose-nanofiber composite sheets (Scheme 1d) were incinerated in air at 450 °C for 6 h (heating speed: 2 °C min^−1^) for the detachment of the primitive cellulose substance and the PDDA ingredient, giving the nanotubular Na_2_MoO_4_/TiO_2_ composites (Scheme 1e) with different contents of Na_2_MoO_4_.

As control experiments, the as-prepared titania/cellulose-nanofiber composite was obtained via the same sol–gel procedure, which was incinerated at 450 °C for 6 h in air (heating speed: 2 °C min^−1^) for the detachment of the primitive cellulose substance, generating the pure titania nanotubes. The pure Na_2_MoO_4_ powder was obtained through the direct calcination of the commercial Na_2_MoO_4_ material at 450 °C for 6 h in air (heating speed: 2 °C min^−1^).

### 2.3. Characterizations

A little sample was dispersed in ethanol by ultrasonic, and the mixture was added onto an aluminum foil, giving the corresponding swatch for the field emission scanning electron microscope (FE-SEM) observation on the Hitachi SU-8010 facilities (acceleration voltage: 5 kV, HITACHI, Tokyo, Japan). In the same way, the mixture was dripped onto a carbon-coated copper grid to generate the swatch for the transmission electron microscope (TEM) on the Hitachi HT-7700 apparatus (acceleration voltage: 100 kV, HITACHI, Tokyo, Japan) and the high-resolution transmission electron microscope (HR-TEM) characterizations. HR-TEM pictures, selected area electron diffraction (SAED) and the energy-dispersive X-ray spectroscopy (EDS) mapping images were gained on a JEM-2100F instrument (JEOL, Tokyo, Japan) working at an acceleration voltage of 200 kV. Powder X-ray diffraction (XRD) measurements were operated on the Philips X’Pert PRO diffractometer (PANalytical B.V., Alemlo, The Netherlands, radiation source: Cu_Kα_, λ = 0.15405 nm, scan rate: 5° min^−1^) in the extent of 10°–90°. The Fourier transform infrared (FT-IR) spectra were acquired by the KBr pellet method on the Nicolet iS10 apparatus (Thermo Fisher Scientific, Waltham, MA, USA). Raman spectra were obtained on the Jobin Yvon LabRam HR UV Raman spectrometer (HORIBA, Paris, France, excitation laser beam wavelength: λ = 532 nm). X-ray photoelectron spectroscopy (XPS) measurements were performed through the VG Escalab Mark II spectrophotometer (VG Instruments, Manchester, UK) with a Mg_Kα_ X-ray source (h = 1253.6 eV). All XPS peaks were calibrated on the basis of the binding energy for the C1s peak of 284.8 eV.

### 2.4. Electrochemical Measurements

The electrochemical measurements were achieved by packaging the typical CR2025 type coin cells (shells purchased from DodoChem, Suzhou, China). The active materials, polyvinylidene fluoride (PVDF) and acetylene black carbon were mingled with a weight ratio of 80:10:10 in N-methylpyrrolidinone (NMP) at the beginning, and then the homogeneous slurry was evenly coated onto the current collector of the nickel foam to prepare the working electrodes. Next, the fabricated electrodes were desiccated (80 °C) for 12 h in a vacuum oven and pressed under 10 atm. The loading mass of the active material on each electrode was maintained at 1–2 mg/cm^2^ approximately. The coin cells were packaged in an Ar gas filled glove box, and the contents of both oxygen and water were below 0.1 ppm. Celgard 2300 microporous polypropylene film (DodoChem, Suzhou, China) and metallic lithium foil were accordingly served as the separator film and counter electrode. The electrolyte was prepared by dissolving LiPF_6_ (1.0 M) in a mixed solution of ethylene carbonate (EC) and dimethyl carbonate (DMC) in a ratio (*v*/*v*) of 1:1. The cyclic voltammetry (CV) curves were obtained on the CHI760D electrochemical work station (CH Instruments, Shanghai, China; sweeping rate: 0.5 mV s^−1^, voltage window: 0.01–3.0 V). The galvanostatic electrochemical cycles were performed at Neware Battery Test System (Neware Technology Co., Ltd., Shenzhen, China), and the tests were implemented at room temperature between 3.0 V and 0.01 V (vs. Li^+^/Li) at various current densities. The electrochemical tests were carried out for at least five times and the results all exhibited good repeatability.

## 3. Results and Discussion

### 3.1. Structural Characterizations of the Nanotubular Na_2_MoO_4_/TiO_2_ Composites

As shown in Scheme 1, the ultrathin titanium dioxide gel film was anchored on the surface of the natural cellulose substance (e.g., commercial ordinary filter paper) via a sol-gel approach in accordance with the reported work [46], and then the double layers (PDDA/Na_2_MoO_4_) were alternatively deposited thereon by the LbL self-assembly manner, yielding the cellulose/titania/(PDDA/Na_2_MoO_4_)_15_ composite sheet. Subsequently, the as-deposited composite sheet was calcined in air for the detachment of the primitive cellulose substance and PDDA ingredient, generating the hierarchically nanotubular Na_2_MoO_4_/TiO_2_ composites, which were formed by the TiO_2_ nanotubes uniformly wrapped with Na_2_MoO_4_ particles. During the preparation processes, through controlling the concentrations of the Na_2_MoO_4_ solutions (0.02, 0.05, 0.1 M), a string of nanotubular Na_2_MoO_4_/TiO_2_ composites with Na_2_MoO_4_ contents of 15.4, 24.1, 41.4% were obtained, which were calculated on the basis of the semiquantitative EDS results (Figure S2). The three nanotubular Na_2_MoO_4_/TiO_2_ composites were accordingly denoted as Na_2_MoO_4_–15.4%-TiO_2_, Na_2_MoO_4_–24.1%-TiO_2_, and Na_2_MoO_4_–41.4%-TiO_2_. In addition, as the concentration of Na_2_MoO_4_ solution was further raised, the content of the Na_2_MoO_4_ component in the sample did not increase evidently.

**Figure 1:**
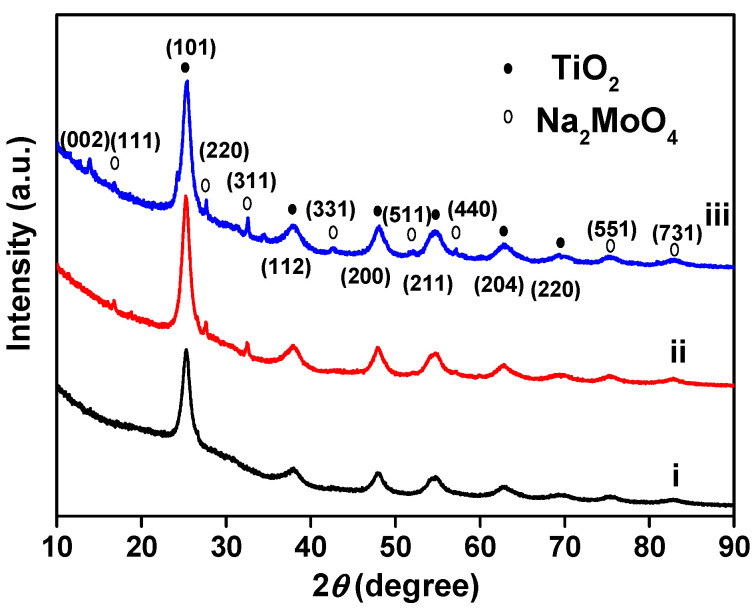
XRD patterns of the nanotubular Na_2_MoO_4_/TiO_2_ composites of (i) Na_2_MoO_4_–15.4%-TiO_2_, (ii) Na_2_MoO_4_–24.1%-TiO_2_, (iii) Na_2_MoO_4_–41.4%-TiO_2_.

Figure 1 presents the powder XRD patterns of a train of nanotubular Na_2_MoO_4_/TiO_2_ composites containing diverse contents of the Na_2_MoO_4_ component. The chief diffraction peaks situated at 2θ = 25.2°, 38.3°, 47.8°, 54.8°, 62.7°, and 69.6° emerged in the XRD patterns of whole samples are assigned to the (101), (112), (200), (211), (204), and (220) crystal planes of the anatase TiO_2_ phase (JCPDS# 71-1169) [49], respectively. For the XRD patterns of the Na_2_MoO_4_–24.1%-TiO_2_ and Na_2_MoO_4_–41.4%-TiO_2_ samples, the diffraction peaks located at 2θ = 16.8°, 27.7°, 32.6°, 43.3°, 52.1°, 57.1°, 75.4°, and 81.0° are discovered, which belong to the characteristic peaks of the *α*-Na_2_MoO_4_ phase (JCPDS# 12-0773), corresponding to the (111), (220), (311), (331), (511), (440), (551), and (731) crystalline planes [21]. However, there are no obvious diffraction peaks of the α-Na_2_MoO_4_ phase detected in the XRD pattern of the Na_2_MoO_4_–15.4%-TiO_2_ sample due to the low content of the Na_2_MoO_4_ component in this sample. The peak at 2θ = 13.9° that detected in the XRD pattern of the Na_2_MoO_4_–41.4%-TiO_2_ sample is attributed to the (002) crystal plane of the Na_2_MoO_4_ 2H_2_O phase in orthorhombic crystalline system [50], which is also found in the XRD pattern of the pure Na_2_MoO_4_ powder (Figure S3). Besides this peak is not discovered in the XRD patterns of the Na_2_MoO_4_–15.4%-TiO_2_ and Na_2_MoO_4_–24.1%-TiO_2_ samples because of the lower content of the Na_2_MoO_4_ component. These XRD analyses keep in accordance with the EDS consequences.

**Figure 2:**
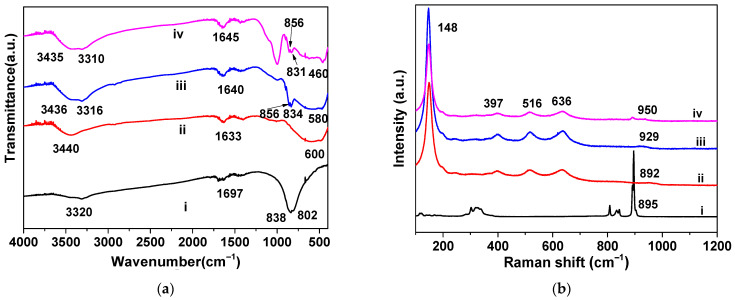
FT-IR spectra (**a**) and Raman spectra (**b**) of the (i) pure Na_2_MoO_4_ powder, (ii) Na_2_MoO_4_–15.4%-TiO_2_, (iii) Na_2_MoO_4_–24.1%-TiO_2_, and (iv) Na_2_MoO_4_–41.4%-TiO_2_ samples.

Figure 2a exhibits the FT-IR spectra of the pure Na_2_MoO_4_ powder and the nanotubular Na_2_MoO_4_/TiO_2_ composites. The bands located at 838–802 cm^−1^ in the spectrum of the pure Na_2_MoO_4_ powder are attributed to the stretching modes of the α-Na_2_MoO_4_ phase [51,52]. For those FT-IR spectra of the Na_2_MoO_4_–41.4%-TiO_2_ and Na_2_MoO_4_–24.1%-TiO_2_ samples, there are vibrational frequencies observed at 856–831 cm^−1^ and 856–834 cm^−1^, which are also assigned to the stretching modes of the α-Na_2_MoO_4_ phase. The slight blue shift of the Na_2_MoO_4_/TiO_2_ composites is due to the interfacial effect of the tensile vibration between the Na_2_MoO_4_ and TiO_2_ phases. However, for the FT-IR spectrum of the Na_2_MoO_4_–15.4%-TiO_2_ sample, there are no obvious peaks that attributed to the α-Na_2_MoO_4_ phase due to the rather low content of the Na_2_MoO_4_ component in this composite. For the Na_2_MoO_4_–15.4%-TiO_2_, Na_2_MoO_4_–24.1%-TiO_2_, and Na_2_MoO_4_–41.4%-TiO_2_ samples, the bands centered at 600, 580, and 460 cm^−1^ are correspondingly assigned to the stretching vibration of Ti−O−Ti in TiO_2_ [53,54,55,56,57]. In addition, the adsorption bands around 3400 and 1640 cm^−1^ for all spectra are indexed to the H−O−H stretching vibration in the molecularly adsorbed H_2_O [53,54,55,56]. The Raman spectra of the nanotubular Na_2_MoO_4_/TiO_2_ composites and the pure Na_2_MoO_4_ powder are revealed in Figure 2b. The bands located at 148, 397, 516, and 636 cm^−1^ in the spectra of the Na_2_MoO_4_/TiO_2_ composites are attributed to the E_g_, B_1g_, A_1g_, and E_g_ modes of the anatase phase TiO_2_ [58]. The obvious peak at 895 cm^−1^ in the spectrum of the pure Na_2_MoO_4_ powder is ascribed to the symmetric stretching modes of MoO_4_^2−^ [59]. Correspondingly, for the spectra of the Na_2_MoO_4_–15.4%-TiO_2_, Na_2_MoO_4_–24.1%-TiO_2_ and Na_2_MoO_4_–41.4%-TiO_2_ samples, the stretching vibration peaks at 892, 929, and 950 cm^−1^ all belong to the symmetric stretching modes of MoO_4_^2−^, which display slight shifts as compared with that of the pure Na_2_MoO_4_ powder, demonstrating the strong interaction between MoO_4_^2−^ and TiO_2_.

As revealed by the XRD, EDS, FT-IR, and Raman results, it has been demonstrated that when the concentration of Na_2_MoO_4_ solution was set as 0.02 M in the preparation processes of the nanotubular Na_2_MoO_4_/TiO_2_ composites, Na_2_MoO_4_ particles were hardly deposited on the titania–cellulose-nanofiber composite, while when the concentrations of Na_2_MoO_4_ solution were set as 0.05, 0.1 M, the final nanocomposites contain various contents of Na_2_MoO_4_. Our previous studies have also proved that the LbL self-assembly method based on the cellulose substance is an effective approach to prepare functional materials with tailored structures [25,47,48].

**Figure 3:**
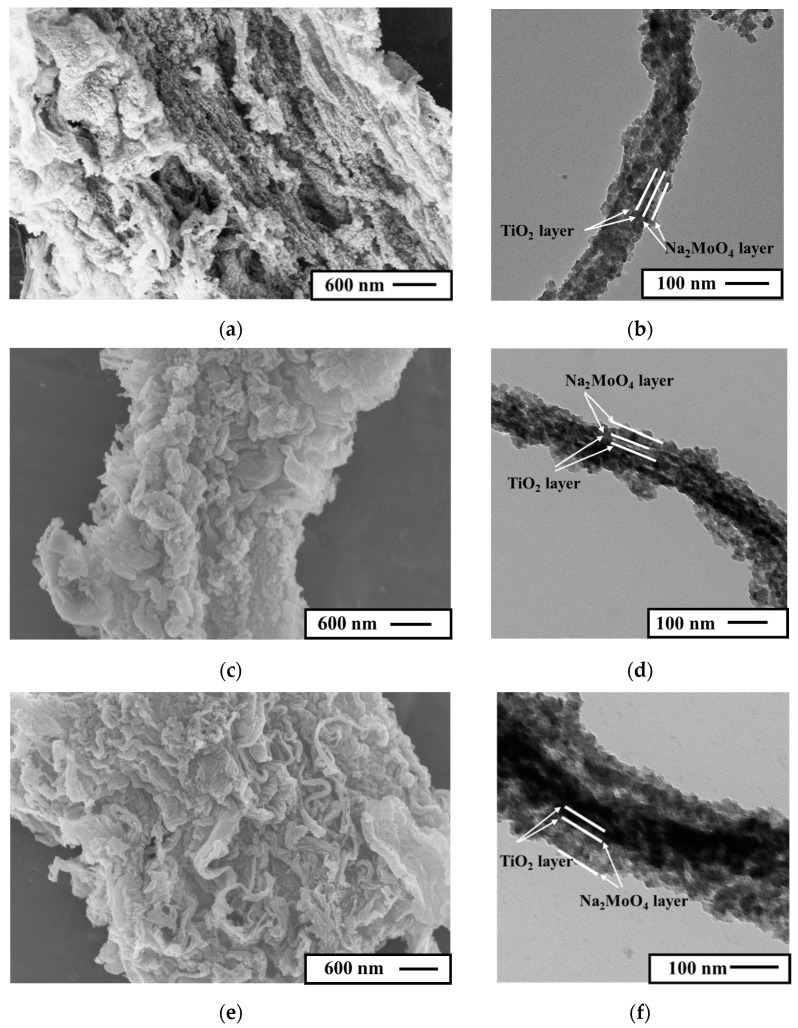
Electron micrographs of the nanotubular Na_2_MoO_4_/TiO_2_ composites derived from natural cellulose substance. FE-SEM images of the (**a**) Na_2_MoO_4_–15.4%-TiO_2_, (**c**) Na_2_MoO_4_–24.1%-TiO_2_, and (**e**) Na_2_MoO_4_–41.4%-TiO_2_ composites, displaying the hierarchical network structures. TEM images of the corresponding individual composite nanotubes of the (**b**) Na_2_MoO_4_–15.4%-TiO_2_, (**d**) Na_2_MoO_4_–24.1%-TiO_2_, and (**f**) Na_2_MoO_4_–41.4%-TiO_2_ composites.

Figure 3 presents the morphologies of the nanotubular Na_2_MoO_4_/TiO_2_ composites. The FE-SEM images of these samples (Figure 3a,c,e) reveal the unique interwoven network structures of the nanotubular Na_2_MoO_4_/TiO_2_ composites that inherited from the premier cellulose substance. It is obvious that these composites are formed by three-dimensional microtubes network, and the microtubes consist of many cross-linked nanotubes with diameters of tens of nanometers, approximately. For the pure Na_2_MoO_4_ powder (Figure S4a), it was formed by the agglomeration of irregular Na_2_MoO_4_ particles. As compared with the nanotubular structure of the pure TiO_2_ sample with smooth nanofiber surface (Figure S4b), the surfaces of these nanotubular Na_2_MoO_4_/TiO_2_ materials are rougher due to the deposition of Na_2_MoO_4_ layers composed of Na_2_MoO_4_ particles. Meanwhile, the deposition of the Na_2_MoO_4_ particles in the nanotubular Na_2_MoO_4_/TiO_2_ composites do not change the initial morphology of the pure TiO_2_ nanotubes. Figure 3b,d,f show the TEM micrographs of the single nanotubes separated from the corresponding Na_2_MoO_4_/TiO_2_ nanocomposites, demonstrating the hollow structures of these composite nanotubes. As compared with the nanotubular structure of the pure TiO_2_ sample (Figure S5b), the composite nanotubes of these Na_2_MoO_4_/TiO_2_ composites are uniformly wrapped with Na_2_MoO_4_ particles. For the pure Na_2_MoO_4_ powder (Figure S5a), it shows micrometer-size agglomeration of Na_2_MoO_4_ particles. As compared with the diameter of the pure TiO_2_ nanotube (ca. 80 nm) (Figure S5b), the diameters of the Na_2_MoO_4_–15.4%-TiO_2_, Na_2_MoO_4_–24.1%-TiO_2_ and Na_2_MoO_4_–41.4%-TiO_2_ composite nanotubes are 94, 100, and 120 nm approximately, indicating the corresponding thicknesses of the Na_2_MoO_4_ layers are about 14, 20, and 40 nm. The SEM and TEM consequences reveal that the contents of the Na_2_MoO_4_ particles in the outer layer of the TiO_2_ nanotubes increase with the enhanced concentrations of the Na_2_MoO_4_ solution in the fabrication processes of the nanotubular Na_2_MoO_4_/TiO_2_ composites, which is in good accordance with the above-mentioned analyses. It is clear that the pure Na_2_MoO_4_ powder sample does not possesses a nanometer-scale structure, and it is easy to aggregate when applied as an anodic material for LIBs. By contrast, these unique three-dimensionally nanotubular structures of the Na_2_MoO_4_/TiO_2_ composites are beneficial to providing more storage sites and shorter Li^+^ diffusion pathways, which are instrumental in the better electrochemical performances.

**Figure 4:**
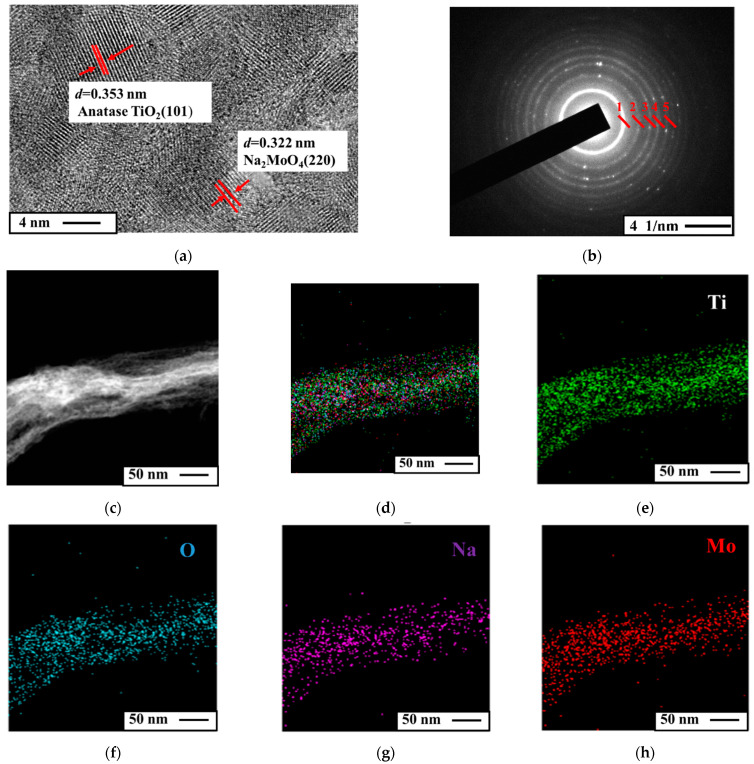
(**a**) HR-TEM image of the nanocomposite tube surface, (**b**) the corresponding SAED pattern, and (**c**–**h**) the EDS elemental mapping of an individual composite nanotube of the nanotubular Na_2_MoO_4_–41.4%-TiO_2_ composite.

Figure 4a shows a HR-TEM image of the composite nanotube surface of the Na_2_MoO_4_–41.4%-TiO_2_ sample, and two types of lattice spacing of 0.353 and 0.322 nm are detected, which correspond to the (101) crystal plane of the anatase phase TiO_2_ and the (220) crystal plane of the *α*-Na_2_MoO_4_ phase, respectively. The diffraction rings labelled by Nos. 1–5 from the inside to the outside in the SAED pattern of the Na_2_MoO_4_–41.4%-TiO_2_ nanocomposite (Figure 4b) correspond to the (101), (004), (200), (211), and (213) reflections of the anatase phase TiO_2_, accordingly; suggesting the conversion of the amorphous titania into anatase nanocrystals during the calcination process [58]. These consequences are in good agreement with the XRD results. The elemental distribution of a single Na_2_MoO_4_–41.4%-TiO_2_ nanocomposite tube is shown in the EDS mapping micrographs (Figure 4c–h), which demonstrate the existence of Na, Mo, O, and Ti elements in this nanocomposite. Moreover, the signals of Ti element are mainly focused on the middle area of the nanotube, while those of the Na and Mo elements are mostly distributed in the outer edges. It further proves that the Na_2_MoO_4_ particles are evenly loaded on the outside of the TiO_2_ nanotubes and the Na_2_MoO_4_–41.4%-TiO_2_ composite duplicates the interwoven network structure of the primitive cellulose substance.

**Figure 5:**
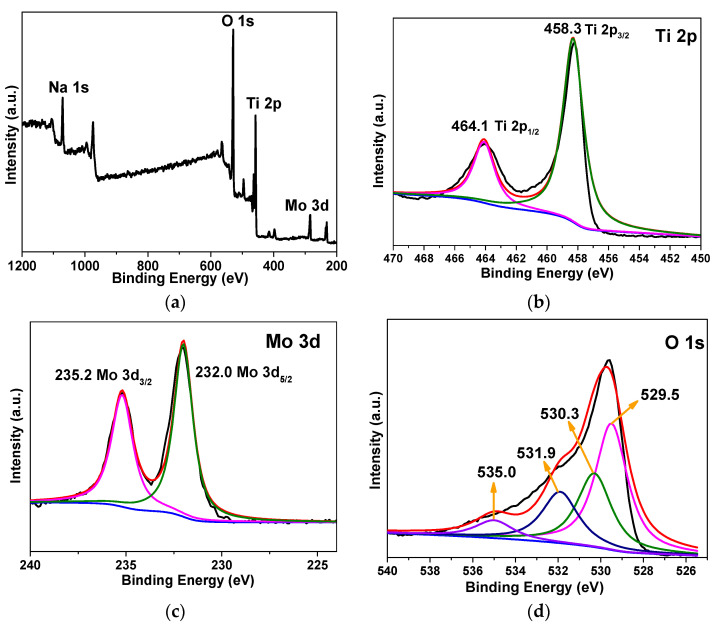
(**a**) Survey XPS spectrum and high-resolution XPS spectra of the (**b**) Ti (2p), (**c**) Mo (3d), (**d**) O (1s) regions of the Na_2_MoO4–41.4%-TiO_2_ nanocomposite.

XPS was employed to determine the chemical states of the species in the Na_2_MoO_4_–41.4%-TiO_2_ composite. Figure 5a presents the overall survey spectrum of this sample, and the peaks of Na (1s), O (1s), Ti (2p), and Mo (3d) are detected. For the high-resolution spectrum of the Ti (2p) region (Figure 5b), the two peaks situated at 458.3 and 464.1 eV with a spin energy separation of 5.8 eV are indexed to Ti (2p_3/2_) and Ti (2p_1/2_) accordingly, demonstrating the main titanium species is the Ti^4+^ of TiO_2_ in this sample [60,61,62]. As exhibited in the high-resolution spectrum of the Mo (3d) region (Figure 5c), the doublet peaks situated at 235.2 and 232.0 eV, with a binding energy gap of 3.2 eV, correspond to Mo (3d_3/2_) and Mo (3d_5/2_), which indicates the existence of Mo^6+^ [63]. Figure 5d performs the high-resolution spectrum of the O (1s) region, and the four peaks situated at 535, 531.9, 530.3, and 529.5 eV are assigned to the physically adsorbed H_2_O molecule, surface hydroxyl group (−OH), lattice oxygen of the Na_2_MoO_4_ phase (Mo−O) and lattice oxygen of the TiO_2_ phase (Ti−O) [64,65,66,67], respectively. These results confirm that the nanotubular Na_2_MoO_4_–41.4%-TiO_2_ composite is composed of Na_2_MoO_4_ and TiO_2_.

### 3.2. Electrochemical Study of the Nanotubular Na_2_MoO_4_/TiO_2_ Composites

Taking all the analyses above into account, the obtained Na_2_MoO_4_/TiO_2_ nanocomposites all persist the three-dimensionally reticular structure of the initial cellulose substance, which consist of TiO_2_ nanotubes uniformly loaded with Na_2_MoO_4_ particles on the tube surfaces. When applied as anodic materials for LIBs, the outer Na_2_MoO_4_ particle layers contribute high capacities of the nanocomposites and the inner TiO_2_ nanotubes are beneficial to buffer the volumetric variation and inhibit the clustering or restacking of the Na_2_MoO_4_ particles throughout the cycling processes, which are vital in enhancing the electrochemical properties of the electrodes.

**Figure 6:**
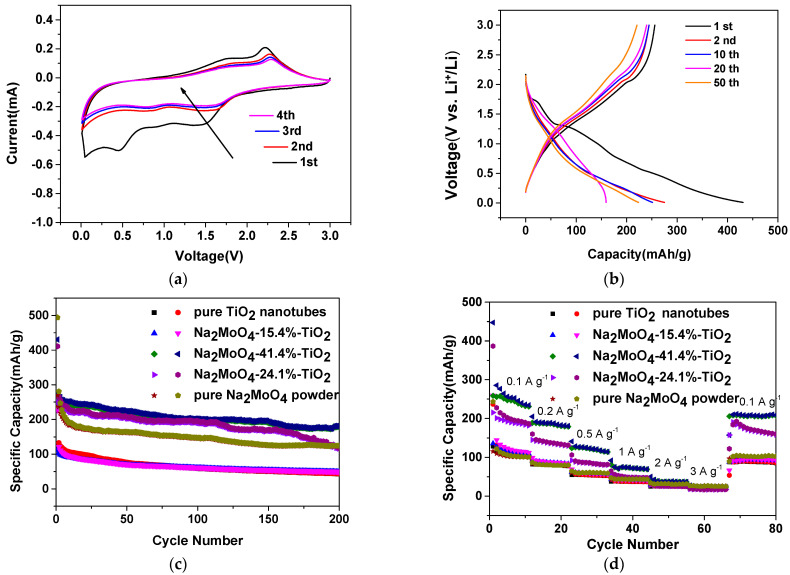
Electrochemical performances of the natural cellulose substance derived nanotubular Na_2_MoO_4_/TiO_2_ composites when employed as anodic materials for lithium-ion batteries. (**a**) Cyclic voltammetry curves of the Na_2_MoO_4_–41.4%-TiO_2_ sample measured at a scan rate of 0.1 mV s^−1^ over the potential window of 0.01–3.0 V vs. Li^+^/Li. (**b**) The charge–discharge profiles of the Na_2_MoO_4_–41.4%-TiO_2_ sample at the 1st, 2nd, 10th, 20th, and 50th cycles under a constant current density of 100 mA g^−1^ between 0.01 and 3.0 V. (**c**) The cycling performances measured at a current density of 100 mA g^−1^ and (**d**) the rate capabilities at various current densities of the nanotubular Na_2_MoO_4_/TiO_2_ composites, pure TiO_2_ nanotubes, and pure Na_2_MoO_4_ powder.

Figure 6a represents the cyclic voltammogram (CV) curves of this nanotubular Na_2_MoO_4_–41.4%-TiO_2_ nanocomposite that recorded at room temperature (voltage: 0.01–3.0 V, scan speed: 0.1 mV s^−1^). The Li storage mechanism of this kind of material belongs to the conversion reaction process, which is analogous to the Li_2_MoO_4_ [19,68] and Na_0_._25_MoO_3_ [69,70,71] materials. During the first discharging process, a small peak at 0.9 V is indexed to the reduction in Na_2_MoO_4_ to metallic Mo and Li_2_O (Equation (2)). A broad cathodic peak observed at 0.5 V belongs to the constitution of the solid electrolyte interface (SEI) layer, which is not detected in the following cycles. After the first discharging process, Na_2_MoO_4_ is completely converted into metallic Mo and Li_2_O. When recharged to 3.0 V, the crystal structure change of the α-Na_2_MoO_4_ phase is non-reversible and the electrode materials become amorphous seriously, which yields an active Na_x_MoO_y_ phase. In the first anodic scan, two peaks at 0.9 and 1.7 V are assigned to the deintercalation of Li^+^, which are corresponding to the conversion from metallic Mo to the amorphous Na_x_MoO_y_ phase [20,72,73]. During the subsequent discharging and charging cycles, the amorphous Na_x_MoO_y_ phase conveys reversible electrochemical reaction with Li, resulting in a Coulombic efficiency of nearly 100% (Figure 6c). Similar redox peaks are observed in the CV plot of the pure Na_2_MoO_4_ powder (Figure S6). In addition, the pair of redox peaks at around 1.5 and 2.20 V in the first CV cycle of this Na_2_MoO_4_–41.4%-TiO_2_ nanocomposite belongs to the insertion and de-insertion process into or from anatase TiO_2_ of Li^+^ (Equation (1)) [49].

(1) $${\mathrm{TiO}}_{2}+{\mathrm{xLi}}^{+}+{\mathrm{xe}}^{-}⇌{\mathrm{Li}}_{x}{\mathrm{TiO}}_{2},$$

(2) $${\mathrm{Na}}_{2}{\mathrm{MoO}}_{4}+6{\mathrm{Li}}^{+}+6e^{-}\to \mathrm{Mo}+{\mathrm{Na}}_{2}O+3{\mathrm{Li}}_{2}O,$$

Figure 6b presents the discharge–charge curves of the Na_2_MoO_4_–41.4%-TiO_2_ electrode in the 1st, 2nd, 10th, 20th, and 50th cycles (current density: 0.1 A g^−1^, voltage: 0.01–3.0 V). For the first discharge curve, a long plateau at ca. 0.5 V is ascribed to the production of the SEI film, and it does not show up in the subsequent cycles. The small plateau appeared at 1.7 V is related to the lithium insertion into titania, while a plateau at ca. 1.2 V is considered to be the lithium insertion into Na_2_MoO_4_. The initial discharge specific capacity of the Na_2_MoO_4_–41.4%-TiO_2_ electrode reaches 430.63 mAh g^−1^ and the charge specific capacity is 255.77 mAh g^−1^, revealing an initial Coulomb efficiency (ICE) of 59.39%. In the first cycle, the large irreversible loss of capacity is a general drawback for the high-capacity metal oxides based anodic materials because of the severe fading of capacity during the processes of lithiation and delithiation, which is assigned to the constitution of the SEI layer aroused by the disintegration of the electrolyte and the irreversible reaction. The discharge capacity in the 2nd cycle declines to 275.04 mAh g^−1^, and the Coulombic efficiency (CE) raises to 88.78%. From the second cycle, the CEs increase quickly and remain as nearly 100%, which represents the excellent cycling durability of the Na_2_MoO_4_–41.4%-TiO_2_ electrode. The discharge capacity is 223.38 mAh g^−1^ with the CE of 98.72% in the 50th cycle, revealing the capacity retention of 51.87% as compared with the initial capacity.

For the pure TiO_2_ nanotubes (Figure S7a), the beginning discharge and charge capacities are 241.31 and 125.45 mAh g^−1^ with a CE of 51.99%. Additionally, it is obvious that the initial discharge and charge profiles of the pure TiO_2_ electrode have characteristic plateaus at 1.70 and 2.2 V, which are ascribed to the lithiation and delithiation of TiO_2_, respectively. In the 50th cycle, the discharge and charge capacities of the pure TiO_2_ electrode quickly fade to 76.43 and 74.42 mAh g^−1^ with a CE of 97.37%, revealing that the discharge retention is only about 31.57% in comparison with the initial capacity. For the pure Na_2_MoO_4_ powder (Figure S7b), the discharge and charge capacities of the first cycle are 471.32 and 219.95 mAh g^−1^, which corresponds to a CE of 46.67%. After the discharge–charge processes for 50 cycles, the discharge and charge capacities of the Na_2_MoO_4_ electrode are 206.82 and 204.25 mAh g^−1^, and the CE is 98.76%. As for the nanotubular Na_2_MoO_4_–15.4%-TiO_2_ and Na_2_MoO_4_–24.1%-TiO_2_ composites, the initial discharge–charge capacities are 226.95 and 112.17, and 410.89 and 249.87 mAh g^−1^, respectively, revealing that the CEs are 49.42 and 60.81%, accordingly. In the 50th cycle, the discharge–charge capacities of the two samples decrease to 70.92 and 69.47, and 207.27 and 203.31 mAh g^−1^, showing the relevant CEs of 97.96 and 98.09%. These results verify the better cycling performance of the nanotubular Na_2_MoO_4_–41.4%-TiO_2_ composite as the anodic material for LIBs, while the increased Na_2_MoO_4_ content in those Na_2_MoO_4_/TiO_2_ nanocomposites has a beneficial effect on enhancing the capacities of the electrodes. Although sodium molybdate has a high theoretical specific capacity, it is prone to appear volumetric change and aggregation during the charge and discharge cycles. Nevertheless, the TiO_2_ nanotubes effectively buffer these changes of the Na_2_MoO_4_ phase as the scaffold, resulting in the extraordinary cyclabilities of those Na_2_MoO_4_/TiO_2_ electrode materials.

The cycling properties of those nanotubular Na_2_MoO_4_/TiO_2_ composites, pure Na_2_MoO_4_ powder and pure TiO_2_ nanotubes as anodic materials (current density: 100 mA g^−1^, voltage: 0.01–3.0 V vs. Li^+^/Li) are displayed in Figure 6c. For the nanotubular Na_2_MoO_4_–41.4%-TiO_2_ composite, its specific capacity decreases rapidly for the first few cycles, and subsequently reaches a considerably steady value (ca. 200 mAh g^−1^) together with a high CE (98%). After repeating the discharge–charge processes for 200 cycles, its reversible specific capacity remains at ca. 180.22 mAh g^−1^ accompanied with a high CE of 99.28%. It is seen that the pure Na_2_MoO_4_ powder show the highest initial capacity of 494.04 mAh g^−1^, but the discharge capacity quickly decreases to 190 mAh g^−1^ after 10 cycles. Moreover, the pure Na_2_MoO_4_ powder gives a reversible specific capacity of only 124.33 mAh g^−1^ after 200 cycles owing to the volumetric variation and clustering of the Na_2_MoO_4_ particles. By contrast, the TiO_2_ nanotubes, Na_2_MoO_4_–15.4%-TiO_2_ and Na_2_MoO_4_–24.1%-TiO_2_ nanocomposites display the reversible specific capacities of 43.70, 49.87, and 117.40 mAhg^−1^ after 200 discharge–charge cycles, accordingly. The nanotubular Na_2_MoO_4_–41.4%-TiO_2_ composite shows the best cycling performances among all the samples because of the alleviation effect of the TiO_2_ nanotubes and the higher content of the Na_2_MoO_4_ component.

The rate performances of those nanotubular Na_2_MoO_4_/TiO_2_ composites, Na_2_MoO_4_ powder and TiO_2_ nanotubes as anodic materials under the current densities of 0.1, 0.2, 0.5, 1, 2, and 3 A g^−1^ were tested and are exhibited in Figure 6d. The stable average discharge capacities of the Na_2_MoO_4_–41.4%-TiO_2_ composite remain at 260, 208, 150, 95, 54, and 30 mAh g^−1^ at the current densities of 0.1, 0.2, 0.5, 1, 2, and 3 A g^−1^, accordingly. It is obvious that when the current density returns to 0.1 A g^−1^, the specific capacity also returns to ca. 210 mAh g^−1^ gradually, and remains stable afterward. In the first 50 cycles, the Na_2_MoO_4_–41.4%-TiO_2_ nanocomposite delivers the best discharge capacity all the time. The discharge capacities of all the samples reduce to be 50 mAh g^−1^ approximately at the current density of 3 A g^−1^. However, the Na_2_MoO_4_–41.4%-TiO_2_ composite still shows the highest discharge capacity as compared with the other samples when the current density returns to 0.1 A g^−1^, revealing the best rate performance.

It is seen from the above results that the higher contents of Na_2_MoO_4_ contained in the Na_2_MoO_4_/TiO_2_ nanocomposites are instrumental in promoting the electrochemical performances of the nanocomposites. Moreover, the robust TiO_2_ nanotubes are also favorable to buffer the strain aroused by the large volumetric variation and inhibit the clustering or restacking of the Na_2_MoO_4_ particles as compared with Na_2_MoO_4_ powder sample. In the researches so far, there were few studies concerning on the preparation of the Na_2_MoO_4_-based materials with specific nanostructures as the anodic materials for LIBs. Additionally, we fabricated the unique hierarchically nanotubular Na_2_MoO_4_/TiO_2_ composite successfully in a simple, highly efficient and environmentally friendly way. Taking the carbon-coated α-Na_2_MoO_4_ nanoplate material with the carbon content of 2.41% that synthesized via a sol–gel method as a comparison, it showed a reversible discharge–charge capacity of 350 mAh g^−1^ after 30 cycles at 30 mAh g^−1^ [21]. Although the hierarchical Na_2_MoO_4_–41.4%-TiO_2_ nanocomposite derived from natural cellulose substance does not possess the superior specific capacity, it delivers more stable cycling performance after more cycles and better rate performance under larger current density, which is highly significant for the practical application of the anodic materials. The excellent electrochemical properties of this nanotubular Na_2_MoO_4_–41.4%-TiO_2_ composite are mainly resulted from the three-dimensionally reticular structure that duplicated from the premier cellulose substance and the cooperative effects between TiO_2_ and Na_2_MoO_4_ components.

## 4. Conclusions

In summary, a bio-inspired nanotubular Na_2_MoO_4_/TiO_2_ composite was prepared with the template of the natural cellulose substance via a layer-by-layer self-assembly approach. The resultant Na_2_MoO_4_/TiO_2_ nanocomposites maintained the three-dimensionally reticular structures of the premier cellulose substance, and the sodium molybdate particles were uniformly loaded outside the titania nanotube surfaces. When applied as anodic materials for LIBs, the nanocomposites performed better electrochemical properties in comparison with the pure Na_2_MoO_4_ powder and pure TiO_2_ nanotubes. The uniformly loaded Na_2_MoO_4_ particles provided high capacity for the Na_2_MoO_4_/TiO_2_ nanocomposites, while the inner TiO_2_ nanotubes served as the buffering matrices to relax the volumetric change and inhibit the aggregating or restacking of the Na_2_MoO_4_ particles. The superior Li^+^ storage properties of the nanotubular Na_2_MoO_4_/TiO_2_ composites were assigned to their hierarchical network structures, together with the cooperative effects between TiO_2_ and Na_2_MoO_4_ components. This strategy sheds an insight on the design of distinct materials with unique structures and functionalities through the layer-by-layer self-assembly route using natural substances as the templates, which are able to be employed in the energy-related fields.

## Data Availability

The data presented in this study are available on request from the corresponding author.

## Supplementary Materials

The following are available online at https://www.mdpi.com/1996-1944/14/2/357/s1, Figure S1: SEM (a) and TEM (b) images of the commercial ordinary filter paper, indicating the hierarchically interwoven network structure and an individual cellulose nanofiber. Figure S2: EDS microanalysis reports of the nanotubular Na_2_MoO_4_/TiO_2_ composites with different Na_2_MoO_4_ contents: (a) Na_2_MoO_4_–15.4%-TiO_2_, (b) Na_2_MoO_4_–24.1%-TiO_2_, (c) Na_2_MoO_4_–41.4%-TiO_2_. Figure S3: XRD pattern of the pure Na_2_MoO_4_ powder. Figure S4: SEM images of (a) the pure Na_2_MoO_4_ powder and (b) the pure TiO_2_ nanotubes samples. Figure S5: TEM images of (a) the pure Na_2_MoO_4_ powder and (b) the TiO_2_ nanotubes samples. Figure S6: The Cyclic voltammetry curves of the pure Na_2_MoO_4_ anodic material measured at a scan rate of 0.1 mV s^−1^ over the potential window of 0.01–3.0 V vs. Li^+^/Li. Figure S7: The discharge–charge voltage profiles of the (a) pure TiO_2_ nanotubes, (b) pure Na_2_MoO_4_ powder, (c) Na_2_MoO_4_–15.4%-TiO_2_ nanocomposite and (d) Na_2_MoO_4_–24.1%-TiO_2_ nanocomposite at the 1st, 2nd, 10th, 20th, and 50th cycles under a constant current density of 100 mA g^−1^ between 0.01 and 3.0 V vs. Li^+^/Li.
