# Supplementary material for: A Bio-Inspired Nanotubular Na2MoO4/TiO2 Composite as a High-Performance Anodic Material for Lithium-Ion Batteries
Source: Materials (Basel). 2021 Jan 13;14(2):357. doi: 10.3390/ma14020357 (PMC7828346; doi:10.3390/ma14020357)
Supplement: Supplementary file 1 [file materials-14-00357-s001.pdf]

materials

MDPI

Supplementary Materials

A Bio-Inspired Nanotubular Na_2MoO_4/TiO_2 Composite as a High-Performance Anodic Material for Lithium-Ion Batteries

Bo Yu, Zehao Lin and Jianguo Huang *

Department of Chemistry, Zhejiang University, Hangzhou 310027, China; 21837072@zju.edu.cn (B.Y.); 11637057@zju.edu.cn (Z.L.)
* Correspondence: jghuang@zju.edu.cn; Tel.: +86-571-8795-1202

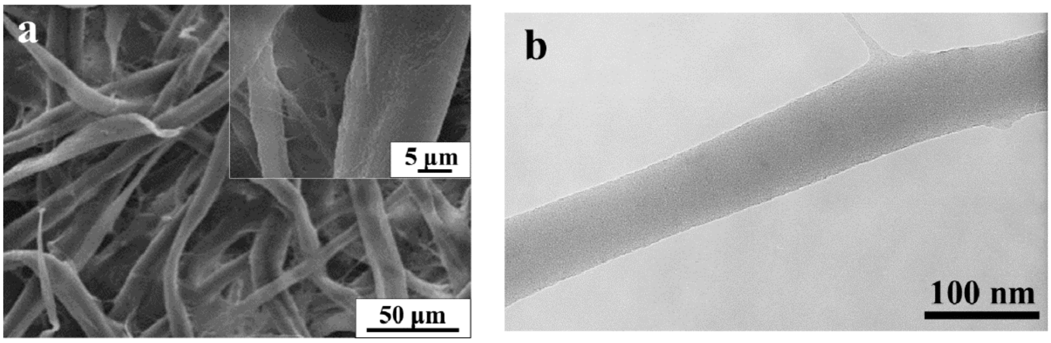

Figure S1. SEM (**a**) and TEM (**b**) images of the commercial ordinary filter paper, indicating the hierarchically interwoven network structure and an individual cellulose nanofiber

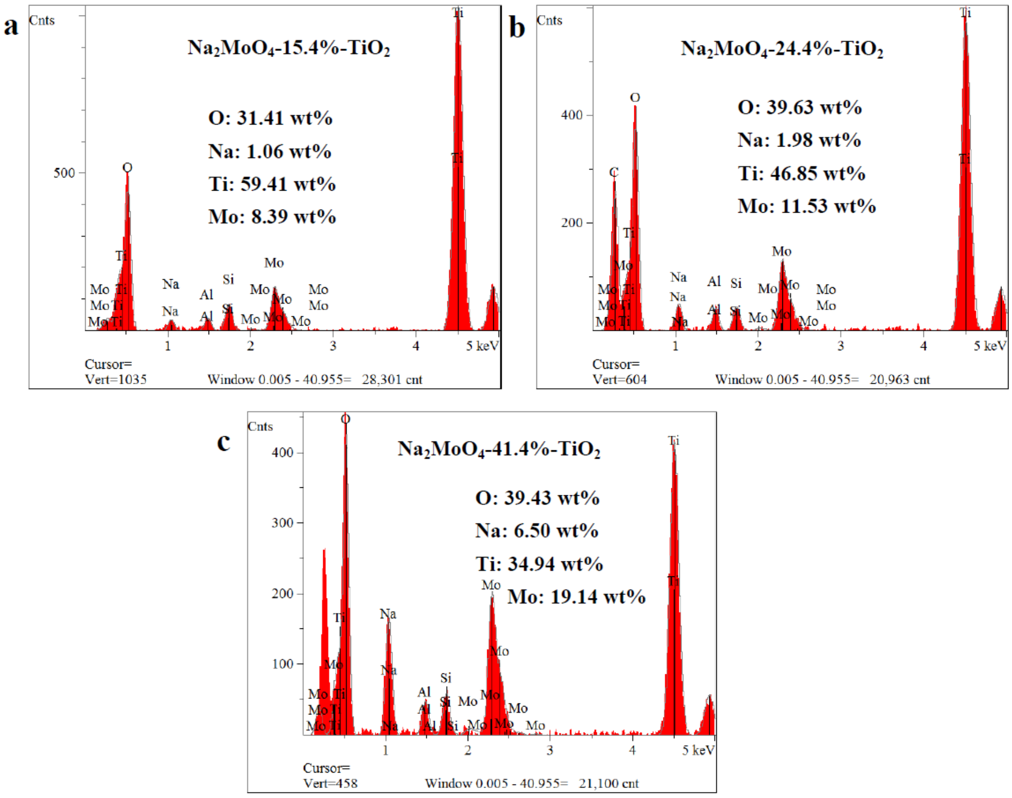

Figure S2. EDS microanalysis reports of the nanotubular Na_2MoO_4/TiO_2 composites with different Na_2MoO_4 contents: (**a**) Na_2MoO_4–15.4%-TiO_2, (**b**) Na_2MoO_4–24.1%-TiO_2, (**c**) Na_2MoO_4–41.4%-TiO_2.

Publisher's Note: MDPI stays neutral with regard to jurisdictional claims in published maps and institutional affiliations.

Copyright: © 2021 by the authors. Licensee MDPI, Basel, Switzerland. This article is an open access article distributed under the terms and conditions of the Creative Commons Attribution (CC BY) license (http://creativecommons.org/licenses/by/4.0/).

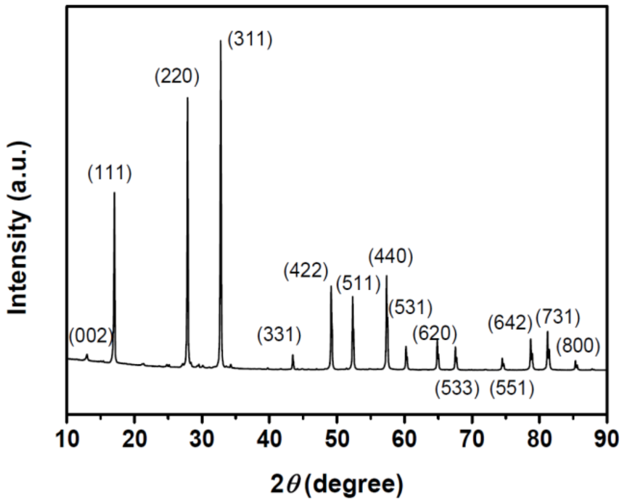

Figure S3. XRD pattern of the pure Na_2MoO_4 powder.

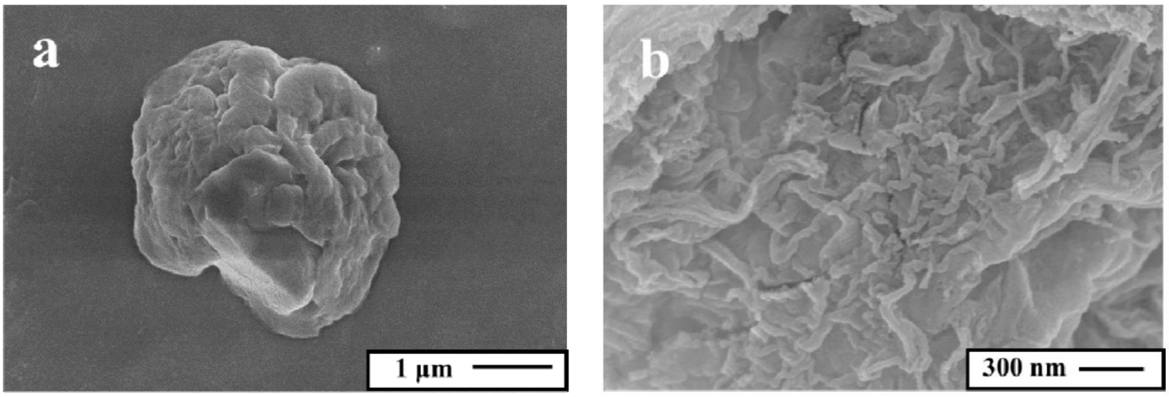

Figure S4. SEM images of (**a**) the pure Na_2MoO_4 powder and (**b**) the pure TiO_2 nanotubes samples.

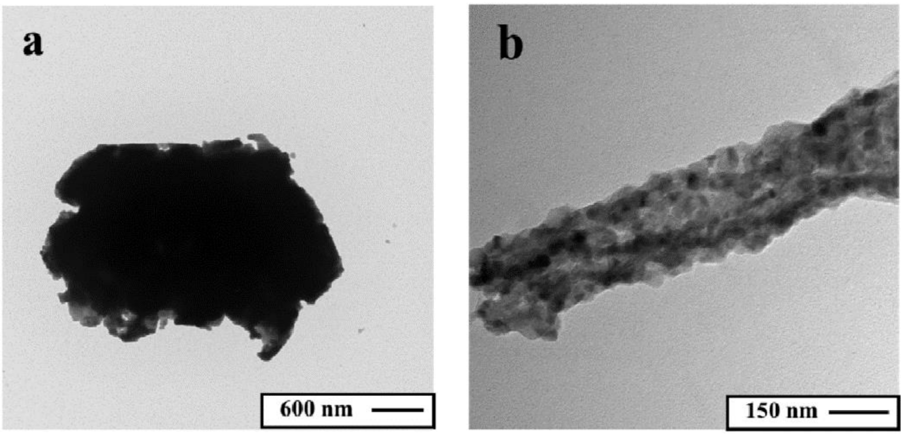

Figure S5. TEM images of (**a**) the pure Na_2MoO_4 powder and (**b**) the TiO_2 nanotubes samples.

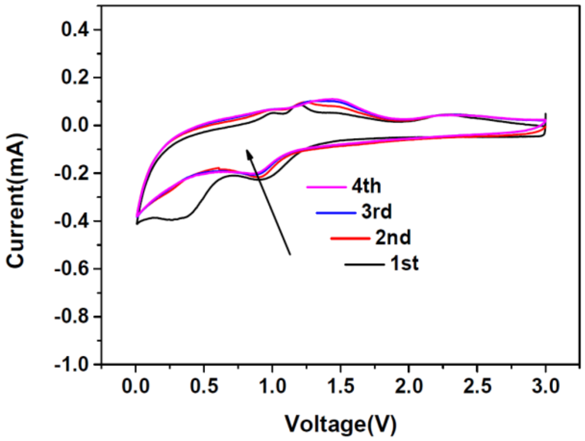

Figure S6. The Cyclic voltammetry curves of the pure Na_2MoO_4 anodic material measured at a scan rate of 0.1 mV s^{-1} over the potential window of 0.01–3.0 V vs. Li^+/Li.

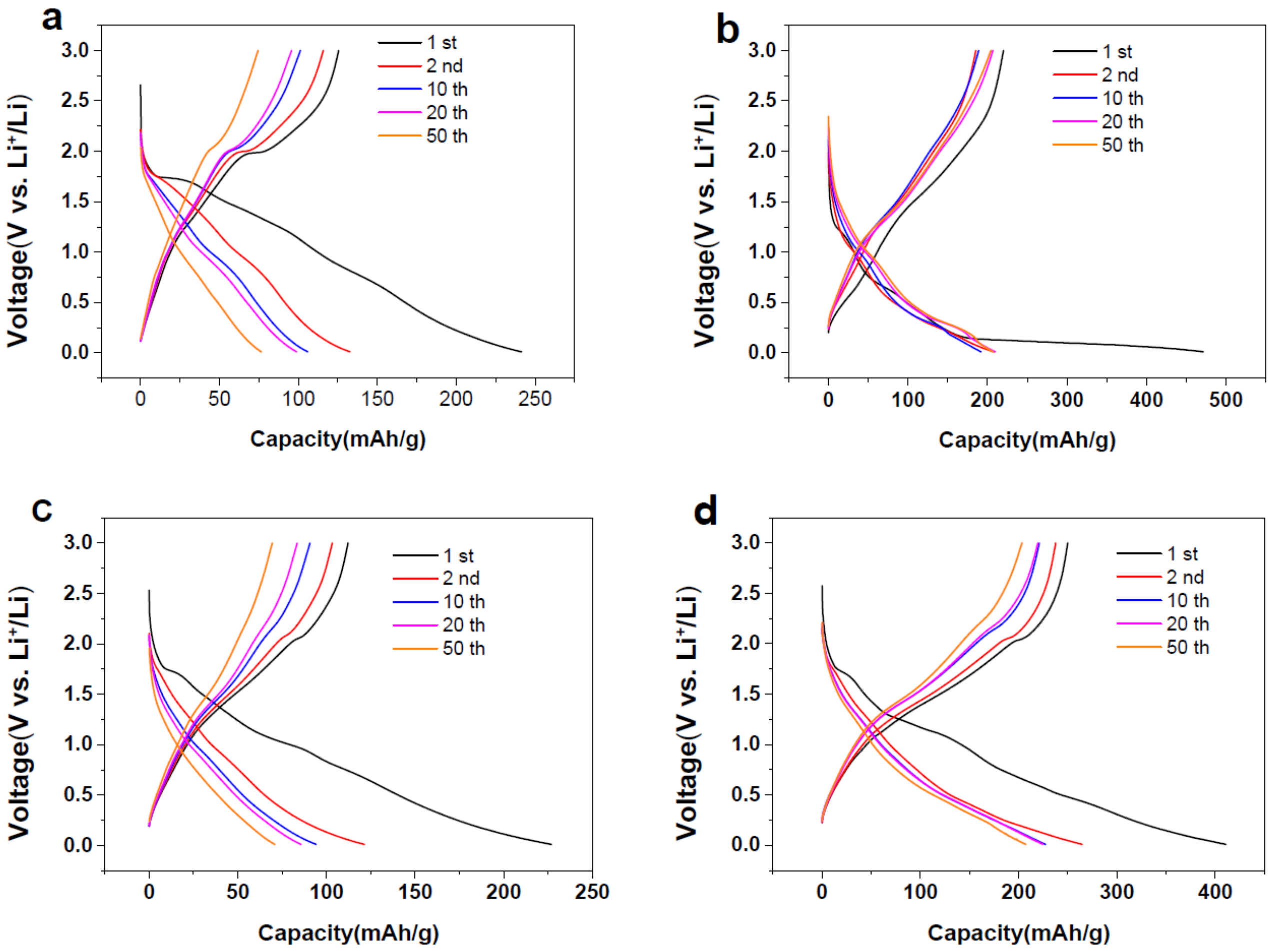

Figure S7. The discharge–charge voltage profiles of the (**a**) pure TiO_2 nanotubes, (**b**) pure Na_2MoO_4 powder, (**c**) Na_2MoO_4–15.4%-TiO_2 nanocomposite and (**d**) Na_2MoO_4–24.1%-TiO_2 nanocomposite at the 1st, 2nd, 10th, 20th, and 50th cycles under a constant current density of 100 mA g^{-1} between 0.01 and 3.0 V vs. Li^+/Li.
